# Development of Polymeric Nanoparticles Loaded with *Phlomis crinita* Extract: A Promising Approach for Enhanced Wound Healing

**DOI:** 10.3390/ijms26052124

**Published:** 2025-02-27

**Authors:** Tahsine Kosksi, Paola Bustos-Salgado, Marwa Rejeb, Arem Selmi, Nawres Debbabi, Lupe Carolina Espinoza, Lilian Sosa, Marcelle Silva-Abreu, Ana Cristina Calpena, Leila Chekir-Ghedira

**Affiliations:** 1 Laboratory of Natural Bioactive Substances and Biotechnology (LR24ES14), Faculty of Dental Medicine, University of Monastir, Av. Avicenne, Monastir 5019, Tunisia; 2 Departament de Farmàcia i Tecnologia Farmacèutica, i Fisicoquímica, Facultat de Farmàcia i Ciències de l’Alimentació, Universitat de Barcelona (UB), Av. Joan XXIII, 27-31, 08028 Barcelona, Spain; 3 Departamento de Química, Facultad de Ciencias Exactas y Naturales, Universidad Técnica Particular de Loja, San Cayetano Alto, Loja 1101608, Ecuador; 4 Institut de Nanociència i Nanotecnologia, Universitat de Barcelona (UB), Av. Diagonal 645, 08028 Barcelona, Spain; 5 Pharmaceutical Technology Research Group, Faculty of Chemical Sciences and Pharmacy, National Autonomous University of Honduras (UNAH), Tegucigalpa 11101, Honduras; 6 Instituto de Investigaciones Microbiológicas (IIM), Facultad de Ciencias, Universidad Nacional Autónoma de Honduras (UNAH), Tegucigalpa 11101, Honduras

**Keywords:** *Phlomis crinita*, PLGA nanoparticles, PLGA-PEG nanoparticles, topical delivery, skin permeation, wound healing

## Abstract

The use of nanoparticles improves the stability, solubility, and skin permeability of natural compounds in skincare products. Based on these advantages, this study aimed to incorporate the *Phlomis crinita* extract into polymeric nanoparticles to improve its topical skin delivery for wound healing purposes. The study involved the preparation of nanoparticles of PLGA and PLGA-PEG (PCE-PLGA-NPs and PCE-PLGA-PEG-NPs) using the solvent displacement method, physicochemical and biopharmaceutical characterization, tolerance studies by the HET-CAM assay and evaluation of skin integrity parameters, and in vitro efficacy via a scratch wound healing experiment. The prepared nanoparticles were nanometer-sized with spherical form and demonstrated an encapsulation efficiency greater than 90%. The major component (luteolin) was released following a kinetic model of hyperbola for PCE-PLGA-PEG-NPs and one-phase exponential association for PCE-PLGA-NPs. Moreover, the important permeability of luteolin skin was observed, especially for PCE-PLGA-PEG-NPs. Both formulations exhibited no irritation and no damaging effects on skin integrity, suggesting their safety. Finally, the results of the scratch wound healing experiment using 3T3-L1 cells revealed significant cell migration and proliferation, with an improved efficacy for PCE-PLGA-PEG-NPs compared to the free extract, demonstrating the potential of this formulation in the treatment of wound healing.

## 1. Introduction

The skin is the body’s largest organ, accounting for about 16% of total body weight. It serves as a crucial protective barrier against various external factors, including physical trauma, chemical exposures, UV radiation, and microbiological threats, such as bacteria and viruses [1]. In cases of injury, the healing of wounds becomes an important and complex process for the survival and overall health of the skin. It is essential to manage wounds properly to promote healing, prevent complications such as infections and scarring, and thereby ensure full recovery from any kind of skin damage [2]. As the focus on health and well-being continues to grow, the pharmaceutical industry has conducted extensive research to develop products that incorporate bioactive compounds for wound healing [3,4]. These compounds are designed to accelerate the healing process and improve skin health, aligning with the broader trend toward enhancing overall well-being.

Natural components have been used for centuries in folk medicine for skincare, but today there is a significant increase in the use of these ingredients in modern formulations. Plant extracts, in particular, are frequently included in skincare products due to their diverse benefits, such as antioxidant and antimicrobial properties, as well as their ability to inhibit tyrosinase activity [5]. These benefits are attributed to the presence of multiple bioactive compounds, such as phenolic acids, flavonoids, and tannins, which have been found to have antioxidant, anti-inflammatory, and antibacterial properties [6].

*Phlomis crinita* is a shrub from the Lamiaceae family, mainly found in Central Asia, Europe, and North Africa [7]. *Phlomis* species were used in folk medicine to treat diseases such as gastric ulcers, diabetes, hemorrhoids, inflammation, and they were mainly used for the healing of wounds. Likewise, their antibacterial activity against *Staphylococcus aureus*, *Escherichia coli*, *Pseudomonas aeruginosa*, *Klebsiella pneumoniae*, and *Aspergillus niger* has been reported [8]. Phytochemical studies have shown that *Phlomis crinita* has high contents of phenolic compounds, mainly flavonoids. Approximately 57 compounds have been identified, with salvianolic acid C, forsythoside B, and luteolin 7-(6″-acetylglucoside) being the substances mostly found in extracts of this plant [9]. Luteolin is an antioxidant agent that inhibits ROS-induced DNA, lipid, and protein damage; it also inhibits enzymes that catalyze the oxidation of cellular components, such as cyclooxygenase and lipoxygenase. In addition, it can protect and increase endogenous antioxidant molecules. For example, superoxide dismutase (SOD), catalase (CAT), as well as glutathione-S-transferase (GST), and glutathione reductase (GR) are known for their antioxidant and anti-inflammatory properties, which affect keratinocyte fibroblasts and various immune cells such as mast cells, neutrophils, or dendritic cells. In allergic contact dermatitis, luteolin in lotion form has shown an ability to reduce redness and increase skin hydration, thus avoiding irritation due to frequent washing and irritants [10,11].

Despite the increasing demand for naturally derived ingredients in skincare, their practical use in cosmetic/dermal therapies often faces various limitations. These limitations include the instability of bioactive compounds during production and storage, low skin permeability, poor solubility, and limited bioavailability that would require repeated application or greater doses, which, in turn, could reduce the drug’s effectiveness for therapeutic usage [12].

In recent years, nanoparticles (NPs) have emerged as a promising solution to overcome these limitations. Many studies have investigated the potential of NPs to improve the stability, solubility, and skin permeability of plant-based ingredients by making use of their distinctive characteristics, such as their small size and large surface area [13,14,15,16]. One of the most used types of NPs are the polymer-based NPs, that can be made from a variety of natural and synthetic polymers, each offering unique properties suitable for different applications. The main synthetic polymers used are the poly (lactic-co-glycolic acid) (PLGA), polycaprolactone (PCL), polymethyl methacrylate (PMMA) and poly (ethylene glycol) (PEG) [16]. These polymers are chosen based on the specific requirements of the application, such as biocompatibility, degradation rate, and the ability to encapsulate and release therapeutic agents effectively. Moreover, they have shown a good affinity for both hydrophilic and lipophilic drugs [17]. Studies have shown that using polymers for NP synthesis is efficient for the encapsulation of plant extracts. This method can enhance the biological activities of plant extracts, such as their antioxidant capacity, improving their antibacterial activity, and modulating the inflammation process [15,18]. Among the polymers used to manufacture NPs as drug release systems, PLGA stands out for its biodegradable properties, whose hydrolysis leads to lactic acid and glycolic acid that are easily metabolized, and thus, minimal systemic toxicity is associated with this polymer [19]. Moreover, PEG combined with PLGA constructs improved systems that increase drug delivery efficacy, mainly for poorly soluble drugs [20]. This combination gives the nanoparticles prolonged circulation and greater permanence in the body thanks to the flexible hydrophilic fraction of PEG [21].

Therefore, the aim of this study was the incorporation of *Phlomis crinita* hydroethanolic extract into polymeric NPs, the characterization of the physical and biopharmaceutical formulations, as well as the determination of their tolerance and the evaluation of their efficacy using a wound healing model.

## 2. Results

### 2.1. Analytical Method Validation

The results of the analytical method used to quantify luteolin 7-(6″-acetylglucoside) from *Phlomis crinita* extract are described in the Supplementary Material. The analysis of the linear regression for each calibration curve exhibited a coefficient of determination (r^2^) greater than 0.99, confirming the method’s linearity (Figure S1). This result was confirmed through a one-way ANOVA test that showed no significant difference between all calibration curves (*p* > 0.999). Moreover, the method exhibited an appropriate sensitivity within the concentration range used (200 to 6.25 µg of luteolin), as it showed low values for LOD and LOQ. The accuracy and precision of the analytical method were assessed at three different concentration levels (maximum, medium, and minimum). The obtained results revealed that the estimated values for relative error (RE) and relative standard deviation (RSD) were below 15%, which is consistent with the acceptance criteria. The determined recovery values further demonstrate the accuracy and reliability of the instrumental method for quantifying the extract. The recovery values fall within the acceptable range (80% to 120%), indicating that the method effectively recovers the analyte and provides accurate measurements. The repeatability study showed that the RSD percentage was approximately 6% (Table S1).

### 2.2. Composition Formula

PCE-PLGA-NPs and PCE-PLGA-PEG-NPs were prepared following the solvent displacement technique achieving the incorporation of 0.1% of *P. crinita* extract. Both formulations showed a homogeneous appearance free of lumps and precipitated particles. Table 1 describes the composition of these formulations.

### 2.3. Physicochemical Characterization

**Figure 1:**
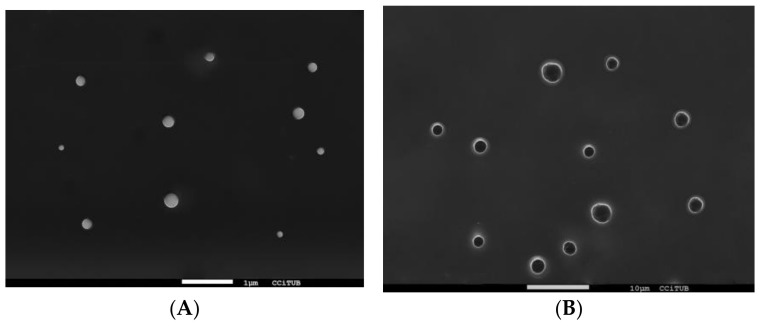
SEM images (**A**) PCE-PLGA-NPs (7500×) and (**B**) PCE-PLGA-PEG-NPs (2000×).

The z-ave of the nanoparticles was 59.5 ± 2.876 nm with a polydispersity index of 0.046 for the PCE-PLGA-NPs and 81.7 ± 0.7584 nm with a polydispersity index of 0.439 for PCE-PLGA-PEG-NPs, indicating reduced size and moderate dispersion. Moreover, both formulations revealed a highly negative zeta potential, with significantly higher values shown for PCE-PLGA-NPs (−38.5 ± 0.379) compared to PCE-PLGA-PEG-NPs (−21.9 ± 1.71). The surface morphology of the NPs evaluated by the SEM technique revealed that both formulations were appropriately distributed and had a spherical morphology (Figure 1). Finally, both formulations exhibited high EE%, with values of 95.335 ± 0.0154% for PCE-PLGA-NPs and 94.977 ± 0.0711% for PCE-PLGA-PEG-NPs.

**Figure 2:**
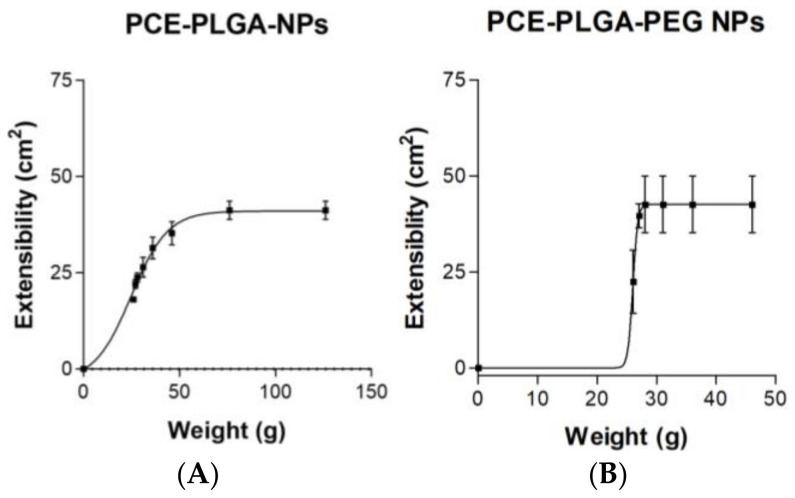
Extensibility profiles of NP formulations. (**A**) PCE-PLGA-NPs and (**B**) PCE-PLGA-PEG-NPs. Data are shown as the mean ± SD, (*n* = 3).

The results of the extensibility presented in Figure 2 revealed a higher but not significant slope and extensibility surface for PCE-PLGA-NPs compared to PCE-PLGA-PEG-NPs, which indicates a higher spreadability for PCE-PLGA-NPs. Moreover, the mathematical treatment of the experimental data revealed that the kinetics of spreadability of both NP formulations followed a Boltzmann sigmoidal profile.

### 2.4. In Vitro Release Studies

**Figure 3:**
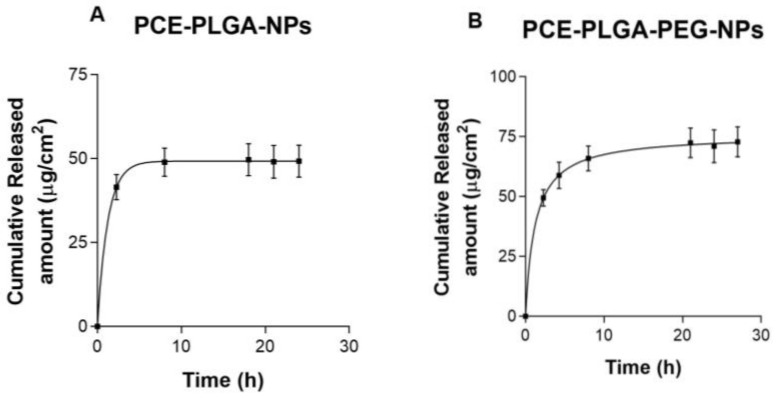
Release profiles of luteolin 7-(6″-acetylglucoside) from NP formulations (**A**) PCE-PLGA-NPs and (**B**) PCE-PLGA-PEG-NPs. The results are presented as the mean ± SD (*n* = 3).

Figure 3 shows the release profiles of luteolin from NP formulations adjusted to different kinetic models. The mathematical model that best fits the experimental data based on the highest coefficient of determination (r^2^) was the one-site binding hyperbola model for PCE-PLGA-PEG-NPs and the one-phase exponential association model for PCE-PLGA-NPs. After 6 h, the release of luteolin from PCE-PLGA-NPs had reached a plateau, whereas PCE-PLGA-PEG-NPs revealed a burst release in the first 2 h, with a sustained release pattern for up to 27 h. The maximum amount of released luteolin was statistically higher for PCE-PLGA-PEG-NPs (75.58 µg/cm^2^) compared to PCE-PLGA-NPs (48.69 µg/cm^2^).

### 2.5. Ex Vivo Permeation Studies

The biopharmaceutical analyses in Table 2 show that the parameters including flow (*J*), the permeability coefficient (*Kp*), and permeated amount at 27 h (Q_27h_) were statistically significantly higher for *ex vivo* permeation studies on injured skin compared to healthy skin for both PCE-PLGA-PEG-NPs and PCE-PLAG-NPs.

**Figure 4:**
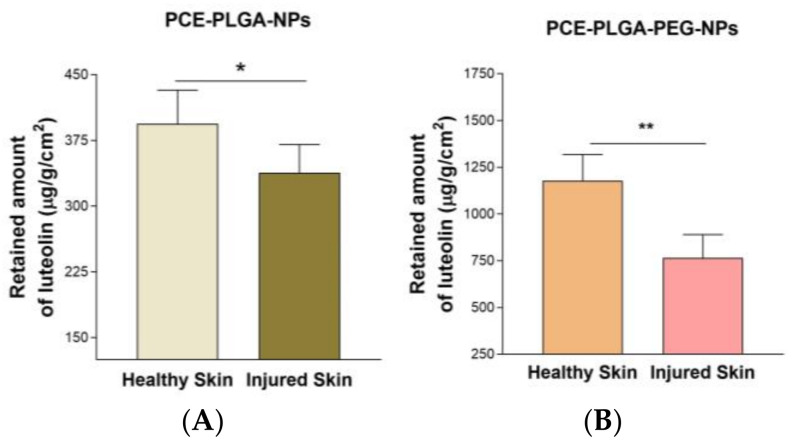
Retained amount of luteolin (μg/g/cm^2^) in the skin. (**A**) PCE-PLGA-NPs and (**B**) PCE-PLGA-PEG-NPs. Results are expressed as the mean ± SD (*n* = 5). Significant statistical differences: * = *p* < 0.05; ** = *p* < 0.01.

Figure 4 shows the different amounts of luteolin retained in healthy and injured skin, which revealed that the PCE-PLGA-PEG-NPs retained higher amounts of luteolin in both healthy and injured skin compared to PCE-PLGA-NPs. Moreover, there was a significantly lower amount of luteolin retained in the injured skin compared to healthy skin for both formulations.

### 2.6. Tolerance Studies

#### 2.6.1. In Vitro Hen’s Egg Test on the Chorioallantoic Membrane (HET-CAM)

**Figure 5:**
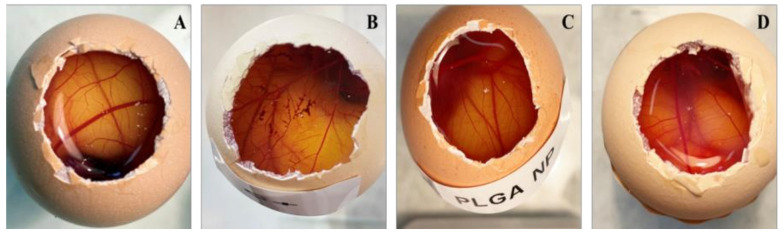
HET-CAM assay egg images of different formulations: (**A**) negative control (saline solution); (**B**) positive control (0.1 N sodium hydroxide solution); (**C**) PCE-PLGA-NPs; and (**D**) PEC-PLGA-PEG-NPs.

**Figure 6:**
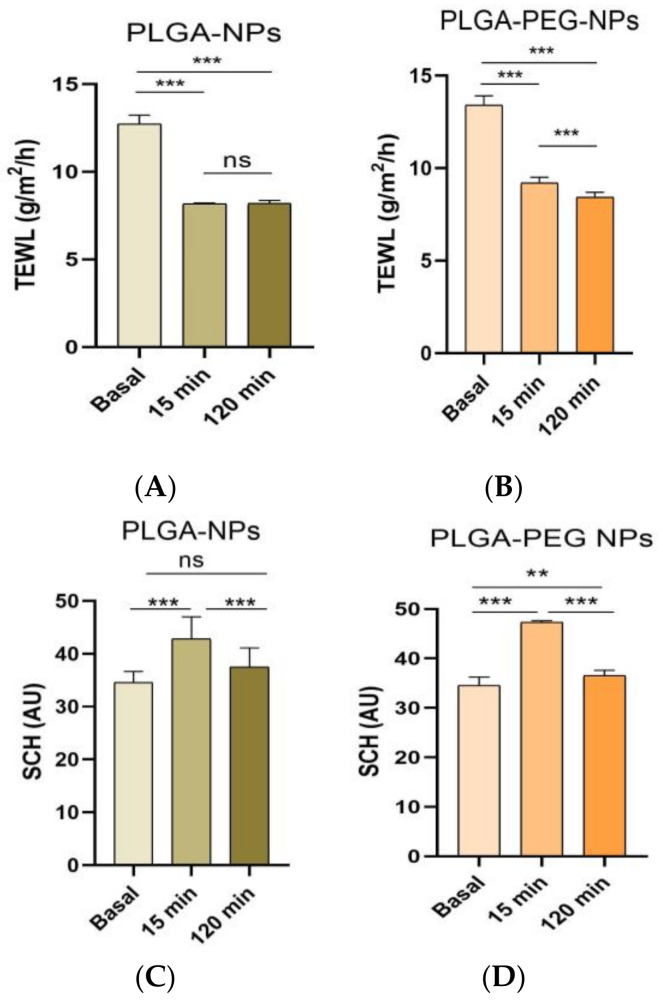
Tolerance studies by the evaluation of the biomechanical parameters after the application of blank formulations. TEWL: transepidermal water loss; SCH: stratum corneum hydration. (**A**) TEWL after the skin application of PLGA-NPs; (**B**) TEWL after the skin application of PLGA-PEG-NPs; (**C**) SCH after the skin application of PLGA-NPs; and (**D**) SCH after the skin application of PLGA-PEG-NPs. Significant statistical differences: ** = *p* < 0.01; *** = *p* < 0.001, ns = non-significant.

The results of the HET-CAM assay (Figure 5) revealed no irritation on the chorioallantoic membrane of fertilized hen’s eggs after 5 min of treatment with PCE-PLGA-PEG-NPs and PCE-PLAG-NPs. These results were similar to the negative control (Figure 5A), while the positive control showed signs of lysis and hemorrhage (Figure 6B). The calculated irritation score (IS) of different formulations is presented in Table 3.

#### 2.6.2. Skin Integrity Parameters

In Figure 6, changes in factors such as Transepidermal Water Loss (TEWL) and Skin Hydration (SCH) before and after using the developed formulations are demonstrated. Data showed a statistically significant decrease (*p* < 0.05) in TEWL values for both NP formulations after 15 min of applying them on the skin.

The stratum corneum hydration (SCH) results revealed a significant increase (*p* < 0.05) 15 min after applying the NP formulations.

### 2.7. Efficacy Studies

#### 2.7.1. Effect of the Free and Encapsulated Extract on Cell Viability

**Figure 7:**
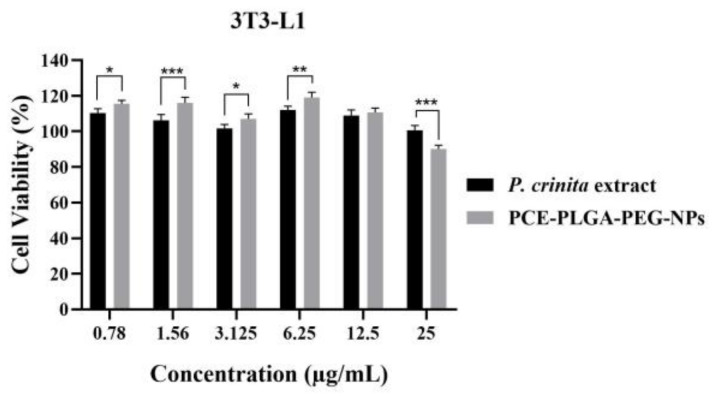
Cell viability percentage of 3T3-L1 fibroblast cells after 24h of treatment with free *P. crinita* extract or PCE-PLGA-PEG-NP formulation. Data shown as the mean ± SD, (*n* = 3). Significant statistical differences: * *p* < 0.05, ** *p* < 0.01, and *** *p* < 0.001.

The MTT test was used to determine cell viability in 3T3-L1 cell lines. As shown in Figure 7, PCE-PLGA-PEG-NPs and the free *P. crinita* extract had no cytotoxic effect on 3T3-L1 cells. In fact, incorporating the extract into the NP formulation improved its effect at lower doses, significantly increasing (*p* < 0.05) cell viability up to 120%, although some concentrations of the NP formulation slightly reduced cell viability. However, until the highest concentration tested (25 µg/mL), the IC50 (half maximal inhibitory concentration) was not attained in the cell line, indicating that the tested PCE-PLGA-PEG-NPs have no cytotoxic or harmful effect.

#### 2.7.2. Scratch Wound Healing Assay

**Figure 8:**
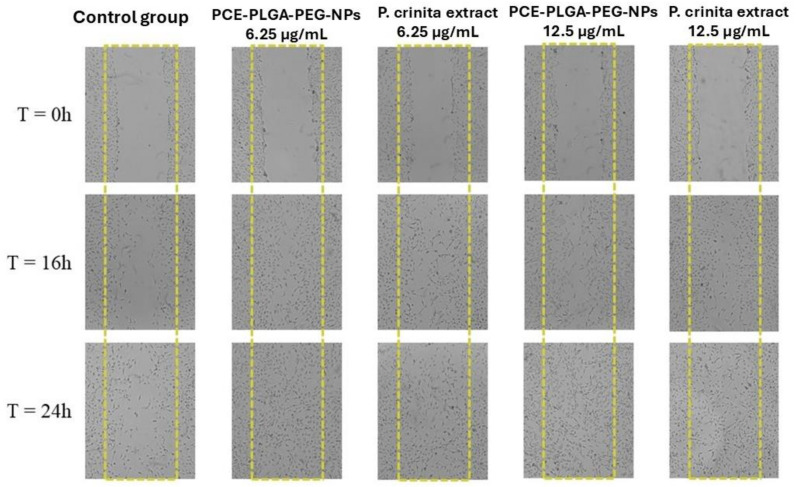
Microscopic images of the wound healing evolution through the scratch assay results conducted using 3T3-L1 fibroblast cells, following treatment with 2 doses of the PLGA-PEG NPs and the free Pc extract (6.25 and 12.5 µg/mL) and the control (CN) at 0, 16, and 24 h.

**Figure 9:**
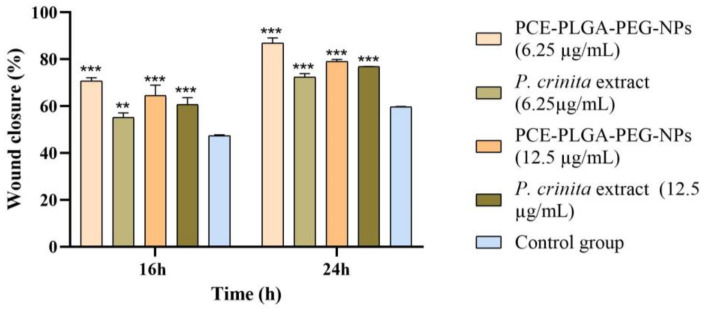
Percentage of wound closure measured from the microscopic images. Data shown as the mean ± SD, (*n* = 3). Significant statistical differences: ** *p* < 0.01 and *** *p* < 0.001.

As shown in Figure 8 and Figure 9, about 59.8% of the wounded area was healed for the control after an incubation period of 24 h, which is likely due to the migration and proliferation of 3T3-L1 cells into the scratched area. On the other hand, the free *P. crinita* extract demonstrated a significantly higher wound closure of 72.4 ± 1.47% at the low concentration (6.25 µg/mL), which increased to 76.9 ± 0.05% for the highest concentration (12.5 µg/mL). This suggests a dose-dependent improvement in wound closure compared to the control group. Interestingly, the PLGA-PEG NP formulation encapsulating *P. crinita* extract significantly enhanced the wound healing potential of the extract at lower concentrations up to 86.90 ± 2.24%, compared to the control and free extract at the same concentration. This suggests that the encapsulation process enhanced the wound healing properties of the extract. At 12.5 µg/mL, the wound closure percentage was slightly higher (79.12 ± 0.85%) compared to the free extract at the same concentration, but it was slightly lower than that observed at 6.25 µg/mL for the same encapsulated extract.

## 3. Discussion

The analytical method used to quantify the main component (luteolin) of the *P. crinita* extract was validated by obtaining a linearity at the concentration range of 200–6.25 µg/mL, as well as the low values of LOD and LOQ, suggesting this method can accurately detect and quantify luteolin at low concentrations. Moreover, the method exhibited favorable accuracy and precision, demonstrating the reliability and suitability of the analytical method and ensuring the generation of precise and consistent results [22].

The incorporation of *P. crinita* extract within two types of polymeric nanoparticles (PLGA and PLGA-PEG NPs) was successfully explored to facilitate the permeability of this extract through the skin, as well as to improve its stability and effectiveness. The nanometric sizes of both formulations, PCE-PLGA-NPs and PCE-PLGA-PEG-NPs, are ideal for topical application. The zeta potential is an effective parameter used to evaluate the stability of particles [23]. The zeta potential results revealed highly negative values, indicating adequate short-time stability [24]. The significant difference in zeta potential observed between PCE-PLGA-NPs and PCE-PLGA-PEG-NPs is probably due to the PEGylation which is known to decrease the negative surface charge as it shields it, resulting in a decline in zeta potential values [23,25]. The morphological structure of the NPs analyzed by SEM imaging revealed a spherical shape. Moreover, the results obtained from the drug loading capacity showed the high encapsulation efficiency of the extract, which confirms the suitability of these polymers to encapsulate natural bioactive compounds.

The in vitro release studies confirmed that luteolin was able to be released from both types of polymeric NPs, and therefore, this parameter will not affect the permeation rate of luteolin through the skin. However, a higher and faster drug release was observed for PCE-PLGA-PEG-NPs. The difference in the release profile could be attributed to the presence of PEG chain moieties at the surface of the PEC-PLGA-PEG-NPs. These molecules have an affinity for water, leading to increased hydration and subsequently their degradation which, in turn, can affect the release characteristics of the encapsulated substance [26].

For topical formulations to exert their pharmacological effect, it is necessary that the incorporated drugs penetrate the stratum corneum, diffuse through the different layers of the skin, and remain in the treated area for an appropriate time to exert their action [27]. PCE-PLGA-PEG-NPs presented higher permeation capacity of luteolin through the skin compared with PCE-PLGA-NPs, showing higher values of flow (*J*), permeability coefficient (*Kp*), permeated amount at 27 h (Q_27h_), and retained amount within the skin (Q_ret_). The high amount of luteolin retained inside the skin from PCE-PLGA-PEG-NPs (Figure 4) favors its local and prolonged effect, which would allow it to reduce the frequency of the administration of the product in clinical practice. This high drug retention and skin permeation of PCE-PLGA-PEG-NPs could be attributed to the characteristics of PEG, which could interact with the intracellular lipids of the stratum corneum, inducing a disruption in their organization and increasing fluidity, which enhances permeation [28]. Previous studies have shown that PEGylation highly improves drug solubility and skin retention compared to other enhancers [29]. The ex vivo permeation study with healthy and injured skin showed higher values for the different permeation parameters, except for Q_ret_ in injured skin compared to healthy skin due to damage to the integrity of the skin, affecting its function as a barrier and making it more susceptible to the passage of substances through it. The higher values of Q_ret_ in the healthy skin suggested that the stratum corneum acts as a reservoir of luteolin [30].

The possible toxicity or irritation of both formulations was assessed by the HET-CAM test, whose results showed that PCE-PLGA-NPs and PCE-PLGA-PEG-NPs had no toxic nor an irritating effect, making them safe for use on the skin and also for periocular application. Additionally, skin tolerance was evaluated by measuring the biomechanical parameters of the skin in healthy volunteers to analyze possible changes in skin integrity after the application of the developed formulations. The volunteers showed no discomfort after the topical application of the products, as they have not experienced any burning sensation, irritation, or itching. The results revealed that the application of PCE-PLGA-NPs and PCE-PLGA-PEG-NPs on healthy skin induced a significant decrease in the TEWL values, along with an increase in the SCH values. These findings are promising in the context of wound healing treatment. Improving the skin barrier function is crucial for optimal wound healing, as it helps create a favorable environment for tissue regeneration, protects against external irritants, and maintains proper and moderate hydration [31].

The first phase in the wound healing process is the inflammatory phase. In this phase, leukocytes infiltrate the wound site and release different mediators, eliminating dead cells. The proliferative phase occurs, subsequently, as a result of the stimulating factors produced by inflammatory cells [32]. Previous studies have demonstrated the potential of *P. crinita* to modulate immune cell function, as well as cytokines and other inflammatory mediators [7]. The evaluation of the in vitro efficacy requires, as a first phase, the evaluation of the possible cytotoxic effect of the formulations studied. In this context, the cytotoxicity effect of the formulations with better biopharmaceutical results (PCE-PLGA-PEG-NPs) was studied using 3T3-L1 cell lines, which are widely used when the compounds tested are intended for topical application [33]. The results of this study revealed that 3T3-L1 cells were able to tolerate the PCE-PLGA-PEG-NPs, as no toxicity was observed during the experiment, demonstrating their cytocompatibility with skin cells. The slight decrease in cell viability at higher concentrations of the formulation may be attributed to enhanced cellular uptake, indicating efficient delivery, since it has been demonstrated that nanoparticle encapsulation increases the local drug concentration within cells, potentially facilitating optimal therapeutic effects through enhanced drug release mechanisms, influenced by the PEG shield interaction with the cell membrane [34,35]. After demonstrating the cytocompatibility of the formulation with the cell lines, its potential as a wound healing agent was evaluated by measuring its impact on cell migration and proliferation. The results of the scratch wound healing experiment using 3T3-L1 cells revealed a significant wound closure with the free extract on fibroblastic cells for both concentrations tested. While higher concentration of the free extract showed an increase in wound closure percentage, suggesting a dose–response relationship, the efficacy of PCE-PLGA-PEG-NPs at the dilution of 6.25 µg/mL outperformed even the highest dose of the free extract. This highlights the benefits of these nanoparticle delivery systems and suggests that the encapsulated form could potentially be more efficient in promoting wound healing. Previous reports have shown that a PEG coating improves nanoparticle stability and interactions with cells because it is able to affect the size and surface charge of nanoparticles, affecting the internalization of the encapsulated compound [36,37]. The enhanced wound healing potential of the polymeric nanoparticles is probably due to the influence of the PEGylation of the PLGA polymer on enhancing the cellular uptake of the plant extract compared to its non-encapsulated form. The role of the *P. crinita* extract in wound healing can be attributed to its luteolin content. The first skin cells that begin to close the wound are fibroblasts, which are stimulated to proliferate and migrate by factors released by hemostatic clots. Several studies using in vitro assays have suggested that luteolin increases the proliferation of fibroblasts [38]. This proliferation then produces molecules, including collagen and fibronectin, to anchor the first layer of the extracellular matrix [39]. The results obtained in this work suggest that the prepared PCE-PLGA-PEG-NPs have great potential for wound healing treatments because they display an enhanced wound healing effect by promoting faster cell migration compared to the free extract and control group.

## 4. Materials and Methods

### 4.1. Materials

PLGA (75:25) was purchased from Sigma Aldrich (Madrid, Spain). Poloxamer 188 was provided by BASF GmbH—BTC Europe (Barcelona, Spain). Diblock copolymer PLGA-PEG 5% (50:50) was purchased from Evonik Corporation (Birmingham, AL, USA). Acetonitrile, glacial acetic acid, dimethyl sulfoxide (DMSO), ethanol, and Tween^®^ 80 were purchased from Panreac (Barcelona, Spain). The dialysis membrane (12 kDa, Dialysis Tubing Visking) was obtained from Medicell International Ltd. (London, UK). DMEM medium, trypsin, streptomycin, penicillin, HEPES, and fetal bovine serum were purchased from Sigma Cell Culture (Courtaboeuf, France). Thiazolyl blue tetrazolium bromide (MTT), a membrane-permeable dye, was obtained from Abcam (Paris, France). A Millipore Milli-Q^®^ water purification system (Millipore Corporation; Burlington, MA, USA) was used. The chemicals and reagents were of analytical grade.

### 4.2. Extract Preparation

*Phlomis crinita* was collected from Jammel, situated in central-eastern Tunisia. The plant was identified according to the flora of Tunisia [40]. Shade-dried and powdered leaves (100 g) were macerated in a hydroethanolic mixture (20% water, 80% ethanol), for 3 days. After filtration, the solvent was evaporated using a rotary evaporator. under reduced pressure at 40 °C; afterward, the extract was frozen at −20 °C and lyophilized to obtain the crude extract.

### 4.3. Quantification of the Luteolin 7-(6″-Acetylglucoside) from Phlomis crinita Extract by High-Performance Liquid Chromatography (HPLC) Analysis

The quantity of luteolin 7-(6″-acetylglucoside) was determined using reversed-phase HPLC-DAD. The HPLC system comprised a Waters^®^ 2695 separation module (Milford, MA, USA) and an Atlantis^®^ C18 5 μm 250 mm × 4.6 mm column. The amount of 7-(6″-acetylglucoside) was detected using a diode array detector Waters^®^ 2996 (Waters, Milford, MA, USA) at a wavelength of 330 nm and a flow rate of 0.6 mL/min under isocratic conditions. The mobile phase consisted of acidified water (glacial acetic acid 5%) and acetonitrile. The injection volume was 50 μL, and a calibration curve was obtained from 6.25 to 200 µg/mL of luteolin 7-(6″-acetylglucoside), dissolved in Milli-Q water. The data analysis was performed using Empower 3^®^ Software (V.7.3.2).

The method used in this study was validated following the standards outlined by the International Conference on Harmonization (ICH). The validation process considered factors such as linearity, sensitivity assessed through the limit of detection (LOD) and limit of quantification (LOQ), precision, accuracy and repeatability.

Linearity was assessed by creating a plot of peak areas against the corresponding nominal concentrations, using a least square linear regression. Additionally, a one-way analysis of variance (ANOVA) test was conducted to confirm the linearity. This statistical analysis was based on comparing the peak areas obtained from each standard with their corresponding nominal concentrations. Statistical significance was established when *p* < 0.05, indicating significant differences.

The limit of detection (LOD) and limit of quantification (LOQ) were used to evaluate the method’s sensitivity. This evaluation is accomplished through linear regression analysis, depending on the standard deviation of the response and the slope of the calibration curve, as previously mentioned [41]. LOD and LOQ are calculated using the following equation:

LOD or LOQ = k × SD/S(1)

*k* is the factor related to the level of confidence (*k* = 3.3 for LOD and 10 for LOQ), SD is the standard deviation of the intercept, and S is the slope.

An inter-day test was carried out to evaluate the accuracy and precision of the analytical method. The test involved analyzing samples at three different concentrations (6.25 µg, 25 µg, and 200 µg) for six consecutive days. The relative standard deviation (RSD, %) of the repeated measurements, which indicated the method’s precision, was calculated using the provided equation. Accuracy was assessed by calculating the relative error (RE, %), which measures the closeness of the measured concentration value to the true value. This method qualifies as accurate and precise if the RE and RSD values are within ±15%.

(2) $$RSD\left(\%\right)=\frac{V_{m\;}-V_{t}}{V_{t}}\;\times \;100$$

(3) $$RE\left(\%\right)=\frac{SD}{M}$$

where RSD is the relative standard deviation, SD is the standard deviation, M is the sample mean, RE is the relative error, $V_{m\;}$ is the measured value, and $V_{t}$ is the true or nominal value.

The instrumental repeatability was determined by performing a repeated analysis of the same sample (200 μg/mL) for 7 consecutive times.

### 4.4. Polymeric NPs Preparation

The polymers used for the experiments have different ratios, and in our preliminary tests with the formulations, we selected the most suitable surfactant for each one, based on physicochemical characterization. PLGA and PLGA-PEG nanoparticles loaded with *P. crinita* extract (PCE-PLGA-NPs and PCE-PLGA-PEG-NPs) were prepared following the solvent displacement technique [42]. A solution of 1 mg/mL of *P. crinita* extract solubilized in ethanol was mixed with a solution of PLGA (90 mg) dissolved in 15 mL of acetone. The stirring process was continued until the complete dissolution of both solutions. Subsequently, the resulting organic phase was added drop by drop, with moderate stirring, to a 50 mL aqueous phase (pH 3.5) containing P188 (10 mg/mL). For the preparation of PLGA-PEG nanoparticles, the method employed was as described previously [43]. An amount of 10 mg of *P. crinita* extract was solubilized in 0.2 mL of DMSO and later mixed with PLGA-PEG dissolved in 5 mL of acetone. The resulting organic solution was then poured slowly into a 10 mL aqueous solution of Tween 80 (2%) under moderate stirring. Afterward, the solvents (ethanol, DMSO, and acetone) were evaporated, and the dispersions of the nanoparticles were concentrated to a final volume of 10 mL under reduced pressure (Büchi R-215V, Flawil, Switzerland).

### 4.5. Physicochemical Characterization

The parameters including the size distribution, zeta potential, morphology, encapsulation efficiency, and extensibility of the formulation were determined. The mean particle size (Z-Ave) and polydispersity index (PdI) of the nanoparticles were measured by photon correlation spectroscopy (PCS) using a Zetasizer Nano ZS (Malvern Instruments, Malvern, UK). The measurements were conducted in triplicate, at 25 °C, after diluting the samples in a 1:10 ratio with Milli-Q water. Additionally, the instrument was used to determine the zeta potential (ZP) of the nanoparticles by Electrophoretic Light Scattering (ELS).

The internal structure of the NP formulations was visualized using Scanning Electron Microscopy (SEM) with a JEOL J-7100F instrument (JEOL Inc., Peabody, MA, USA). The samples were subjected to carbon coating using an Emitech K950X coater (Quorum Technologies Ltd., Kent, UK) which also enhanced sample conductivity.

To determine the encapsulation efficiency, PCE-PLGA-NPs and PCE-PLGA-PEG-NPs were subjected to filtration/centrifugation, using a centrifugal filter with a molecular weight cutoff (MWCO) of 100 kDa (Vivaspin 500, Satorius, Göttingen, Germany). A dispersion of 0.5 mL of NPs were centrifuged at 14,000 rpm for 20 min (Sigma 301K 8 centrifuge, Osterode am Harz, Germany). Subsequently, the filtrate obtained from the centrifugation process was analyzed using a reversed-phase HPLC-DAD to quantify the amount of unencapsulated luteolin 7-(6″-acetylglucoside) contained in the *P. crinita* extract.

The encapsulation efficiency (EE%) was calculated using the following equation:

(4) $$EE(\%)=\left[\frac{Total\;amount\;of\;luteolin-Free\;luteolin}{Total\;amount\;of\;luteolin}\right]\times 100$$

To assess the extensibility of the NP formulations, a glass plate weighing 26.06 g was collocated on top of the 0.3 mL of formulations. Afterwards, a series of weights (1, 2, 5, 10, 20, 50, and 100 g) were added on top of the glass plate at 1 min intervals. The study was conducted in triplicate at room temperature. For each applied weight, the diameter (cm) of the resulting circle was measured, and the corresponding increase in surface area (cm^2^) was calculated as a function of the progressively increasing weights.

### 4.6. In Vitro Release Studies

The in vitro release assessment of the NP formulations was conducted using amber vertical Franz diffusion cells (FDC 400, Crown Glass, Somerville, NY, USA) with an effective diffusional area of 2.54 cm^2^. Prior to being placed in the Franz cell, a dialysis membrane (12 kDa, Dialysis Tubing Visking, Medicell International Ltd., London, UK) was hydrated for a period of 24 h. The receptor medium used was Milli-Q water, which was constantly stirred at 700 rpm to maintain sink conditions at 32 ± 1 °C. A volume of 0.3 mL of formulations was placed in the donor compartment. At predetermined intervals, samples (0.3 mL) were drawn out of the receptor compartment using a syringe and replaced with an equal volume of Milli-Q water at the same temperature. As previously mentioned, samples were examined using HPLC-DAD and a UV detector. The acquired data, expressed as the mean ± SD (*n* = 3), were fitted to various mathematical models, such as the one-phase exponential association and one-site binding (hyperbola) models, in order to determine the release kinetics. The model that provided the best r^2^ value was chosen.

### 4.7. Ex Vivo Permeation Studies

Human skin samples were obtained during an abdominal lipectomy of a healthy 38-year-old woman (Hospital of Barcelona, SCIAS, Barcelona, Spain), in accordance with the Ethical Committee of the Hospital of Barcelona (number 002, dated 17 January 2020). These samples were cut into 0.5 µm-thick sections using a dermatome (Model GA 630, Aesculap, Tuttlingen, Germany). The collected skin pieces were then stored at −20 °C. This experiment was carried out using healthy and injured skin. To injure the skin, a microneedle was used by simply rolling it over the skin surface. Franz cells with a 0.64 cm^2^ diffusion area were used in the skin permeation experiment. The skin samples with the stratum corneum facing the upper compartment were placed between the donor and the receptor chamber. A volume of 0.2 mL of the formulation was applied on the skin surface in the donor chamber. Milli-Q water was filled in the receptor compartment, which was kept at 32 ± 1 °C using a circulating water jacket under continuous stirring (700 rpm). At predetermined intervals, samples of 200 µL were withdrawn from the receptor medium and replaced with an equal volume of Milli-Q water at the same temperature. HPLC-DAD was used to quantify the amount of luteolin 7-(6″-acetylglucoside). Five replicates of this experiment were carried out.

Permeation Parameters

To determine the flux (µg/cm^2^/h) through the skin, the cumulative amount of luteolin 7-(6″-acetylglucoside) permeating the skin was plotted against time. The linear portion of the curve was analyzed using linear regression analysis performed with Prism^®^, V.3.00 software (GraphPad Software Inc., San Diego, CA, USA). The slope of this linear regression analysis was divided by the diffusion area to calculate the flux.

The permeability coefficients (Kp, cm/h) were obtained by dividing the flux (J) by the initial drug concentration (C0) in the donor compartment.

(5) $$Kp=\frac{J}{C0}$$

Following the permeation experiment, the skin was cleaned using a sodium lauryl sulfate solution (0.05%), rinsed with distilled water, and allowed to dry. The skin area in direct contact with formulations was cut and weighed. The extract content retained was extracted with Milli-Q water in an ultrasonic bath for 50 min. The obtained solutions were assessed using HPLC-DAD in order to determine the amount of luteolin 7-(6″-acetylglucoside) retained (Q_ret_, μg/g skin/cm^2^) in the skin.

### 4.8. Tolerance Studies

#### 4.8.1. In Vitro Hen’s Egg Test on the Chorioallantoic Membrane (HET-CAM)

The HET-CAM (Hen’s Egg Test—Chorioallantoic Membrane) is a test employed to evaluate the potential irritation caused by formulations after periocular application. Ten-day embryonated hen’s egg (supplied by the G.A.L.L.S.A. farm, Tarragona, Spain) were incubated in a climatic chamber at 37.0 ± 0.5 °C and 58% relative humidity. The eggshell and the inner membrane were removed in order to reveal the chorioallantoic membrane (CAM). The different samples (300 µL) were then applied directly to the CAM with a positive control (0.1 N solution of NaOH) and a negative control (0.9% NaCl). The assays were performed in triplicate using different eggs for each substance. The eggs were monitored for 5 min to assess the presence of hemorrhage (H), lysis (L), and coagulation (C). The Irritation Index (IS, irritation score) was calculated using the following equation:

(6) $$IS=\left[\frac{\left(301-hemorrhage\;time\right)}{300}\times 5\right]+\left[\frac{\left(301-lysis\;time\right)}{300}\times 7\right]\;+\left[\frac{\left(301-coagulation\;time\right)}{300}\times 9\right]$$

The drug is classified as non-irritating for IS values between 0 and 0.99; slightly irritating for scores between 1.0 and 4.99; moderately irritating for scores between 5.0 and 9.99; and extremely irritating for scores between 10.0 and 21.0.

#### 4.8.2. Skin Integrity Parameters

Transepidermal water loss (TEWL) is a measure of the quantity of water lost through the stratum corneum (SC) by diffusion, which represents an important indicator of skin barrier function. TEWL was measured using a Tewameter^®^ TM 300 (Courage-Khazaka electronic GmbH, Cologne, Germany). The measurement was conducted in the forearm (basal levels) of 10 volunteers before and after applying the blank formulations at different time intervals (5, 15, 30 min, 1.30 h, and 2 h). This experiment was carried out in a climate-controlled room (25 ± 2 °C, relative humidity 45%). Prior to the measurements, the volunteers had 30 min to adapt to the climate.

The hydration level of the outermost layer of the skin, known as the stratum corneum (SCH), was assessed using the Corneometer^®^ CM 825 (Courage-Khazaka electronic GmbH, Cologne, Germany). This device employs a capacitance method to measure skin hydration by analyzing the influence of the water content on the skin’s electrical properties. Measurements were taken at various time intervals as follows: baseline (before treatment) and at 5, 15, 30 min, 1.30 h, and 2 h after the application of the blank formulations. The data, presented as arbitrary units, are reported as the mean ± SD (*n* = 10).

### 4.9. Efficacy Studies

#### 4.9.1. Cell Cultures

3T3-L1 fibroblasts were used in the experiments. 3T3-L1 were cultured in Dulbecco’s modified Eagle’s medium (DMEM) supplemented with 10% FBS, 1% antibiotics (100 IU/mL penicillin and 100 µg/mL streptomycin), and 2.5% HEPES.

#### 4.9.2. Effect of the Free and Encapsulated Extract on Cell Viability

3T3-L1 cells (5 × 10^3^ cells/well) were seeded in 96-well plates and incubated for 24 h at 37 °C with 5% CO_2_. After incubation, the cells were treated with different concentrations (25, 12.5, 6.25, 3.125, 1.56, and 0.78 µg/mL) of either free extract or the formulation with better biopharmaceutical results diluted in culture medium. Cells treated with DMEM were used as the control. After 48 h of incubation, 50 µL of MTT (1 mg/mL) was added to each well, and the plates were incubated for 3h at 37 °C. Finally, the MTT was removed, and the formazan crystals were dissolved with 100 μL of DMSO. The absorbance was spectrophotometrically measured at 550 nm using a ThermoScientific (Vantaa, Finland) plate reader.

#### 4.9.3. Scratch Wound Healing Assay

The scratch wound healing experiment was carried out as previously described [44]. 3T3-L1 cells (5 × 10^5^ cells/well) were seeded in 6-well plates and incubated at 37 °C for 24 h. Cell layers were scratch-wounded with a sterile pipette tip, treated with the free extract and the selected formulation, and incubated at 37 °C for 24 h. Images were captured with an inverted DM-IRBE microscope (Leica, Rueil-Malmaison, France) and analyzed using image analysis software (ImageJ v 1.54). The wound closure % was evaluated in comparison to control cells.

### 4.10. Statistical Analysis

Two-way ANOVA was used for the multi-group comparison, followed by the Tukey post hoc test. Meanwhile, to compare two groups, Student’s *t*-test was performed. Data are presented as the mean ± standard deviation and the statistical significance was established at *p* < 0.05 using GraphPad Prism 8.0.2 (263) (GraphPad Software, San Diego, CA, USA). ImageJ software was used to analyze the images.

## 5. Conclusions

This study developed polymeric nanoparticles (PLGA and PLGA-PEG) encapsulating *Phlomis crinita* extract to enhance wound healing, achieving high encapsulation efficiency, nanometric size, and stability. The PLGA-PEG nanoparticles exhibited superior drug release and skin permeability, particularly on injured skin, and they promoted significant wound closure by enhancing fibroblast migration and proliferation, outperforming the free extract. Both formulations demonstrated excellent safety profiles, with no irritation or cytotoxicity, while maintaining skin barrier integrity and improving hydration. These findings highlight the potential of nanoparticle-based systems to overcome the limitations of natural bioactive compounds, such as poor stability and permeability, offering an effective and safe approach for advanced topical wound care.

## Figures and Tables

**Table 1:** Composition formula of PCE-PLGA-NPs and PCE-PLGA-PEG-NPs.

Components	PCE-PLGA-NPs (%)	PCE-PLGA-PEG-NPs (%)
*P. crinita* extract	0.1	0.1
PLGA	0.9	×
PLGA-PEG	×	0.95
P188	1	×
Tween 80	×	2
Water	98	96.95

The organic fraction used to solubilize the drug and polymer was evaporated (see Section 4.4). × = Not included.

**Table 2:** Estimated permeation parameters of NP formulations. Values are reported as the mean ± SD (*n* = 5).

Biopharmaceutical Parameters	Formulations			
	PCE-PLGA-NPs		PCE-PLGA-PEG-NPs	
	Healthy Skin	Injured Skin	Healthy Skin	Injured Skin
*J* (μg/h/cm^2^)	0.838 ± 0.041	1.128 ± 0.372	1.349 ± 0.167	2.097 ± 0.092
*Kp* × 10^−4^ (cm/h)	8.38 ± 0.41	11.3 ± 3.72	13.5 ± 1.67	21.0 ± 0.92
Q_27h_ (μg)	20.56 ± 1.28	19.93 ± 1.75	27.80 ± 2.67	38.49 ± 0.02

Abbreviations: *J* (flux), *Kp* (permeability coefficient), and Q_27h_ (permeated amount at 27 h).

**Table 3:** Irritation score of PLGA NP and PLGA-PEG NP formulations (mean ± SD of *n* = 3).

	Negative Control	Positive Control	PCE-PLGA-NPs	PCE-PLGA-PEG-NPs
Irritation score (IS)	0.07 ± 0.00	7.14 ± 1.26	0.07 ± 0.00	0.07 ± 0.00

## Data Availability

The data presented in this study are available in this article.

## Supplementary Materials

The following supporting information can be downloaded at: https://www.mdpi.com/article/10.3390/ijms26052124/s1.
