# Supplementary material for: Development of Polymeric Nanoparticles Loaded with Phlomis crinita Extract: A Promising Approach for Enhanced Wound Healing
Source: Int J Mol Sci. 2025 Feb 27;26(5):2124. doi: 10.3390/ijms26052124 (PMC11901007; doi:10.3390/ijms26052124)
Supplement: Supplementary file 1 [file ijms-26-02124-s001.zip › ijms-3469427-supplementary.pdf]

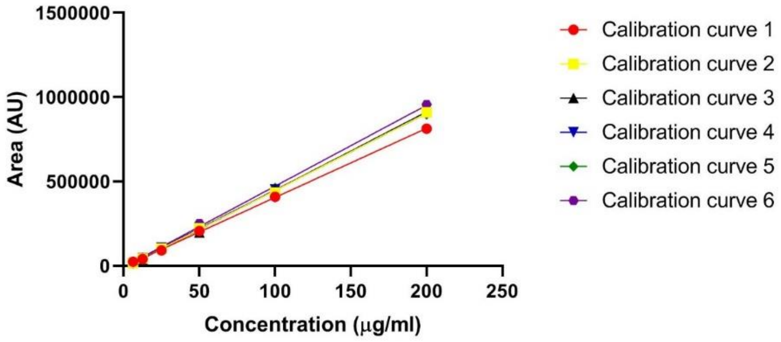

Figure *S1*. Linearity of the analytical method

Table *S1*. Assessment of linearity, LOD, LOQ, accuracy and precision (at three concentration levels), and repeatability of the HPLC analytical method for quantification of luteolin contained in *P. crinita* extract.

Parameters	Linearity		LOD	LOQ	Accuracy		Precision	R.I.S
	r^2	*p*-value	Mean ± SD (µg/ mL)		RE (%)	Recovery (%)	RSD (%)	RSD (%)
					200 (µg/mL)<br>25 (µg/mL)<br>6.25 (µg/mL)			200 (µg/mL)
	0.9995	0.999	3.47 ± 1,39	10.52 ± 4,21	0.472<br>4.214<br>13.392	99.96<br>99.63<br>101.13	-0.044<br>-0.372<br>1.125	5.98

r^2 = coefficient of determination at 200–6.25 (µg/mL). LOD = limit of detection; LOQ = limit of quantification; RE = relative error; RSD = relative standard deviation; R.I.S = repeatability of instrumental system.
